# Population-, sex- and individual level divergence in life-history and activity patterns in an annual killifish

**DOI:** 10.7717/peerj.7177

**Published:** 2019-06-27

**Authors:** Eli S.J. Thoré, Arnout F. Grégoir, Bart Adriaenssens, Charlotte Philippe, Robby Stoks, Luc Brendonck, Tom Pinceel

**Affiliations:** 1 Animal Ecology, Global Change and Sustainable Development, KU Leuven, Leuven, Belgium; 2 Institute of Biodiversity, Animal Health and Comparative Medicine, College of Medical, Veterinary and Life Sciences, University of Glasgow, Glasgow, United Kingdom; 3 Systemic Physiological and Ecotoxicological Research, University of Antwerp, Antwerp, Belgium; 4 Evolutionary Stress Ecology and Ecotoxicology, KU Leuven, Leuven, Belgium; 5 Water Research Group, Unit for Environmental Sciences and Management, North-West University, Potchefstroom, South Africa; 6 Centre for Environmental Management, University of the Free State, Bloemfontein, South Africa

**Keywords:** *Nothobranchius*, Behavior, Rhythmicity, Diurnal, Pace-of-life, Animal personality, Killifish, Life history, POLS

## Abstract

Variation in life-history strategies along a slow-fast continuum is largely governed by life-history trade-offs. The pace-of-life syndrome hypothesis (POLS) expands on this idea and suggests coevolution of these traits with personality and physiology at different levels of biological organization. However, it remains unclear to what extent covariation at different levels aligns and if also behavioral patterns such as diurnal activity changes should be incorporated. Here, we investigate variation in life-history traits as well as behavioral variation at the individual, sex and population level in the Turquoise killifish *Nothobranchius furzeri*. We performed a common garden laboratory experiment with four populations that differ in pond permanence and scored life-history and behavioral (co-) variation at the individual and population level for both males and females. In addition, we focused on diurnal activity change as a behavioral trait that remains understudied in ecology. Our results demonstrate sex-specific variation in adult body size and diurnal activity change among populations that originate from ponds with differences in permanence. However, there was no pond permanence-dependent divergence in maturation time, juvenile growth rate, fecundity and average activity level. With regard to behavior, individuals differed consistently in locomotor activity and diurnal activity change while, in contrast with POLS predictions, we found no indications for life-history and behavioral covariation at any level. Overall, this study illustrates that diurnal activity change differs consistently between individuals, sexes and populations although this variation does not appear to match POLS predictions.

## Introduction

Life-history strategies vary among individuals, populations and species as a consequence of natural selection in response to ecological conditions (Stearns, 1976; Hill & Kaplan, 1999). Traditionally, trade-offs between life-history traits (e.g., maturation time, fecundity and body size) are at the core of life-history theory (Stearns, 1976; Stearns, 1989). These trade-offs result in a slow-fast continuum with late maturing, slow growing organisms with a long lifespan and delayed reproduction on the one end and early maturing, fast growing organisms with a short lifespan and precocious reproduction on the other (Réale et al., 2010). Such patterns can result from differential investment in immediate versus future reproductive output as part of alternative life history strategies (Dammhahn et al., 2018).

Recently, the pace-of-life hypothesis was expanded by including suites of coevolving life-history, physiological and behavioral traits (i.e., pace-of-life syndrome hypothesis, POLS) (Ricklefs & Wikelski, 2002; Réale et al., 2010; Adriaenssens, 2017). Specifically, behavioural and physiological traits that have a functional role in mediating life-history trade-offs were included (e.g., traits related to resource acquisition and risk-taking) (Dammhahn et al., 2018). Fast, ‘proactive’ life styles are expected to be associated with high activity, high boldness and a high metabolism while slow phenotypes exhibit low activity, shyness and a low metabolism (see Réale et al. (2010) for a full overview). There is some support for this concept at the interspecies, inter-breed and population levels such as in domesticated dogs where variation in personality and life-history is constrained to a continuum between long-living, shy, breeds and more bold breeds with a shorter lifespan (Careau et al., 2010). This trait covariation has been assumed to either reflect a common underlying regulatory mechanism (e.g., pleiotropy) or correlational selection that drives co-evolution of traits by favoring trait combinations with the highest fitness advantage (Réale et al., 2010).

In addition to stimulating the study of behavioral traits (e.g., spontaneous activity) as essential components of the POLS framework, Réale et al. (2010) also suggested to apply the POLS to between-individual variation (Careau et al., 2008; Réale et al., 2010). Despite support for the POLS at higher levels of biological organization (Wikelski et al., 2003; Wiersma et al., 2007), evidence to extend the concept to individual level differences has been mixed (Debecker et al., 2016; Adriaenssens, 2017; Dammhahn et al., 2018). In a recent meta-analysis, Royauté et al. (2018) evaluated 179 cases of how POLS applies to the integration of behaviors with physiology and life-history. Overall, this meta-analysis yielded very weak support for POLS, especially for vertebrates. To date, the incidence of POLS is not always fully understood, and it often remains unclear what mechanism underlie covariation patterns at different levels of biological organization (Réale et al., 2010; Adriaenssens, 2017; Dammhahn et al., 2018). The heuristic framework of POLS should be fine-tuned and, to this end, multi-level approaches could further elucidate how life-history variation is generated and maintained. In the current study, natural populations of annual killifish are used to investigate life-history and behavioral (co-)variation at multiple levels of biological organization and to test the idea that activity patterns are an integral component of POLS.

*Nothobranchius* killifish are interesting model organisms to study the evolution of life-history variation, including variation in behavior (Cellerino, Valenzano & Reichard, 2015). Fish of this African genus have an exceptionally short life cycle as an adaptation to the ephemeral nature of the temporary ponds they inhabit (Polačik, Blažek & Reichard, 2016). Killifish are limited by the length of the wet phase to complete the aquatic stage of their life cycle and shorter inundation lengths drive selection for fast maturation (Terzibasi et al., 2008; Terzibasi et al., 2013; Cellerino, Valenzano & Reichard, 2015). Upon maturation, *Nothobranchius* species produce drought-resistant eggs that remain dormant in the sediment to bridge dry periods and that hatch at the onset of the rainy season when ponds start to flood (Cellerino, Valenzano & Reichard, 2015). A fraction of these eggs will arrest development and hatch at a later inundation to prevent complete reproductive failure in case of an unsuitable inundation (Pinceel et al., 2015).

**Figure 1:**
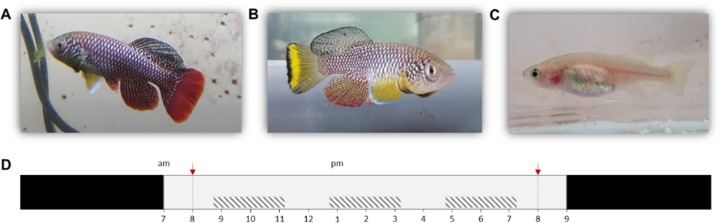
*Nothobranchius furzeri* and the daily schedule during the observation period. (A, B) Conspicuously colored *N. furzeri* males, red and yellow morph respectively, and (C) female *N. furzeri*. (photographs by Arnout F. Grégoir). (D) Fish were kept at a 14 h: 10 h day:night regime (light period from 7am–9pm). Red arrows indicate the administration of live food (*Chironomus* larvae). Shaded boxes indicate the three daily sample bursts. Time of day is indicated for the light period.

Especially *Nothobranchius furzeri* Jubb 1971 (Turquoise killifish) is now a commonly used model in various fields of biological research as it exhibits the shortest captive life span recorded for vertebrates (typically 5–6 months) (Terzibasi et al., 2008). Although individuals from all known populations have a relatively short lifespan, extensive intrinsic variation in lifespan between populations has been recorded. This variation likely evolved in relation to time stress that is associated with the length of the growing season (i.e., wet phase) of the source habitat, with populations from ponds with a shorter wet phase (in dry regions) exhibiting a shorter lifespan compared to populations from ponds with more relaxed time stress conditions (in more humid regions) (Cellerino, Valenzano & Reichard, 2015; Blažek et al., 2016). Thus, pond permanence is a major environmental determinant of the lifecycle of killifish (Grégoir et al., 2017; Grégoir et al., 2018). In the laboratory, dormant *Nothobranchius* eggs can be easily stored and allow for synchronized hatching after a developmental period that can be manipulated in length by the researcher, ranging from 2 weeks to multiple years (Polačik, Blažek & Reichard, 2016). Next to a short lifespan and fast maturation, *N. furzeri* is also a naturally bold species. This is illustrated by its risk-taking behavior in non-novel situations compared to other fish (model) species. Finally, the species is characterized by a clear sexual dichromatism and dimorphism (Figs. 1A–1C). Combined, these are desirable characteristics of a model organism for behavioral studies (Polačik, Blažek & Reichard, 2016; Thoré et al., 2018a; Thoré et al., 2018b).

Although *Nothobranchius* species have been proposed as promising model organisms for studying the effects of senescence on the circadian system (Lucas-Sánchez et al., 2014), variation in diurnal activity changes among individuals and populations remains to be studied. Recently, diurnal variation in activity has been suggested as a novel dimension of animal personality (Alós, Martorell-Barceló & Campos-Candela, 2017). Temporal segregation facilitates the co-existence of competitors or serves as a mechanism of predator avoidance (Kronfeld-Schor & Dayan, 2003). For instance, Norway rats (*Rattus norvegicus*) have been found to shift their nocturnal foraging activity to diurnal activity to minimize predation by nocturnal red foxes (*Vulpes vulpes*) (Fenn & MacDonald, 1995). Also, Alanärä, Burns & Metcalfe (2001) demonstrated that foraging activity of dominant individuals of the brown trout (*Salmo trutta*) peaks at moments that are optimal to maximize food intake and when predation risks are minimal. In contrast, activity of subordinate individuals peaks at alternate moments to minimize competition with stronger individuals and differences in daily activity patterns increase with an increasing intensity of intraspecific competition. There is growing evidence for the adaptive value of circadian or diurnal activity changes (Yerushalmi & Green, 2009) and such changes have been shown to impact ecological processes such as predation, foraging efficiency and resource acquisition or competition. Still, intra- and interpopulation variation in activity changes is rarely studied and taken into account in ecology (Randler, 2014; Závorka et al., 2016).

Several researchers have suggested to include diurnal activity as a trait in POLS. Their case is supported by the fact that individuals or populations might differ consistently in diurnal activity change (see Alós, Martorell-Barceló & Campos-Candela, 2017) and that diurnal activity change is often correlated with life-history traits (Miyatake, 2017; Matsumura, Ryohei & Miyatake, 2019). Furthermore, such changes can impact fitness by mediating, for instance, reproductive success (Beaver et al., 2002; Yerushalmi & Green, 2009), Finally, if not a consequence of POLS, consistent individual differences in diurnal activity pattern seem unlikely as it would be more beneficial to adjust activity patterns in response to the situation at hand (indefinite plasticity) or an ‘optimal’ diurnal activity pattern would be universally selected for.

While spontaneous activity and behavioral traits in general have been addressed in literature, most studies are biased towards male animals. Therefore, potential effects of sex on behavioral patterns such as diel rhythmicity are neglected (Kuljis et al., 2013; Krizo & Mintz, 2015). Such effects are likely since a large number of traits are sex dependent including morphology, physiology, life-history (Shaw, Kokko & Neubert, 2017), mating behavior (Killen et al., 2015) and ecology (e.g., predation sensitivity) (Han, Jablonski & Brooks, 2015). Consequently, studies should take into account sex (Beery & Zucker, 2011). Although males and females typically differ in their energetic investment in different life-history traits and despite well-studied sex-specific differences in life-history strategies, so far sex-specificity of POLS has been largely ignored. This calls for theoretical and empirical studies on sex-specific differences in the POLS framework (Niemelä et al., 2012; Debecker et al., 2016; Dammhahn et al., 2018; but see Hämäläinen et al., 2018). According to Bateman’s principle, males are assumed to benefit from allocating more energy to mating rate whereas females allocate more energy to egg production and longevity. Debecker et al. (2016) applied this principle to damselflies and formulated the expectation that sexes would diverge along a fast-slow continuum, with males and females positioned at the fast and slow side of the spectrum respectively. Given that sex-specific POLS patterns are expected to be ubiquitous, a recent surge of scientific interest has been directed to this issue (Hämäläinen et al., 2018; Immonen et al., 2018; Tarka et al., 2018). However, further empirical work to test these predictions is needed (Dammhahn et al., 2018).

Blažek et al. (2016) contrasted two natural populations of *N. furzeri* (Nf222 and Nf121, see Table 1) and confirmed that the population originating from a more arid region (i.e., short wet phase) had a shorter lifespan compared to the population from a more humid region (i.e., longer wet phase). However, in contrast to predictions of the POLS this was not associated with divergence in growth rate, maturation time and active behavior at the interpopulation level. In the current study, we considered multiple levels of biological organization and investigated whether average activity levels and diurnal changes in activity of *N. furzeri* differ among individuals, sexes and populations originating from ponds that differ in pond permanence within the framework of the POLS. For this, we observed activity of a total of 121 *N. furzeri* male and female individuals from four naturally-derived populations that were selected to cover variation in pond permanence (short-lived ponds vs. long-lived ponds, associated with short fish life expectancy vs. long life expectancy, respectively). In addition, we included the homozygous *N. furzeri* laboratory strain (GRZ) as a fifth group to compare this commonly used strain in killifish research with natural populations (Supplemental Information) (Cellerino, Valenzano & Reichard, 2015; Polačik, Blažek & Reichard, 2016). By also monitoring juvenile growth rate, maturation time, fecundity and adult body size of individual fish, we were able to assess correlation between life-history and behavior (including diurnal activity change), both at the individual and population level. The potential of diurnal activity change as a candidate-trait for life-history covariation in a POLS framework is explored and discussed.

**Table 1:** Overview of the studied populations of *N. furzeri* and their respective origins.

Population collection code	Region	Latitude	Longitude	Altitude (m)	Water depth^a^ (cm)	Pond size^b^ (m^2^)	AAP^c^	PET^d^	Aridity index^e^
NF2	Wet	−24.06	32.73	77	20–60	75–250	576	1,823	0.3205
NF121	Wet	−24.36	32.97	30	40–70	900–4,800	638	1,797	0.3439
NF222	Dry	−21.87	32.80	158	15–50	40–300	545	1,809	0.3087
NF414	Dry	−22.55	32.73	97	15–40	375–1,250	464	1,823	0.2543

**Notes.**

NF222 and NF414 originate from the relatively arid region along the Chefu river in central Mozambique, while NF2 and NF121 originate from the humid region along the Limpopo river in southernmost Mozambique.

AAP average annual precipitation (mm) PET potential evapotranspiration (mm/month) aridity index degree of water deficiency due to dryness of the climate, calculated as the ratio of average annual precipitation over potential evapotranspiration (a lower index indicates a more arid region)

a,b Personal communication with Radim Blažek (Institute of Vertebrate Biology, Academy of Sciences of the Czech Republic).

c,d,e Data from WorldClim—Global Climate Data (http://www.worldclim.org).

We hypothesize that, contrary to populations from long-lived ponds, populations from short-lived ponds, in which pond drying restricts the window for growth and reproduction, show early maturation, precocious reproduction and fast growth supported by a consistently high activity level throughout the day (i.e., fast pace-of-life). We expect these patterns of covariation also to be present among individuals within populations as hypothesized by Réale et al. (2010), where individuals are expected to differ in life-history and behavior along a fast-slow pace-of-life continuum. Given the strong sexual dimorphism in both size and coloration, and consistent with the predictions of Debecker et al. (2016), we expect to find sex-specific differences in life-history strategy and in behavior, with males and females positioned at the fast and slow end of the pace-of-life continuum, respectively.

## Materials and Methods

### Fish breeding and source habitats

We used a total of five *N. furzeri* populations, of which four natural-derived *N. furzeri* populations (see Table 1) that originate from habitats along an aridity gradient (see Terzibasi et al., 2008) (see Supplemental Information for results on the homozygous laboratory strain GRZ, *n* = 24). Two populations (NF222 and NF414, *n* = 28 and *n* = 24 respectively) originate from a relatively arid region from central Mozambique where inundations are typically short (i.e., short life expectancy). This is supported by datalogger measurements which were used to estimate pool inundations from 2011 to 2012 (Terzibasi et al., 2013). The two other populations (NF2 and NF121, *n* = 27 and *n* = 18 respectively) originate from a more humid region in southern Mozambique, where inundations last longer (i.e., longer life expectancy) (Terzibasi et al., 2008). From here onwards, these habitats will be referred to as short-lived and long-lived ponds, respectively. All populations were reared for at least three generations under optimal common garden conditions in the lab prior to the start of the experiment.

Fish were hatched in 2L tanks by submerging eggs and peat in dechlorinated tap water (7.8 pH, 600µS/cm conductivity) at a temperature of 14 °C—method based on Polačik, Blažek & Reichard (2016). Two days post-hatching, fish larvae were transferred to individual 250 mL glass jars to allow individual monitoring. Starting at an age of four days up until one month, individual fish were housed in 1L plastic, transparent jars after which they were individually transferred to 2L plastic, transparent tanks until the end of the experiment. Each tank was provided with a small piece of filter wool (Belcopet NV, Brugge, Belgium) to sustain necessary microbiota for a good water quality. During the experiment, fish were kept in a climate-controlled room at a constant air temperature of 24 °C (water temperature 21.6 °C ± sd 0.75) and a 14 h: 10 h day:night regime. Twice per week, tanks were cleaned and the water was refreshed to ensure a constant and good water quality. Tanks were repositioned in a random fashion after every cleaning session to prevent site-specific habituation whilst allowing for visual contact between individuals of both sexes.

Fish larvae were fed twice a day (8am and 5pm) an *ad libitum* quantity of *Artemia fransiscana* nauplii (Ocean Nutrition, Essen, Belgium). After three weeks their diet was supplemented with small live *Chironomus* larvae (Aquaservice, Antwerp, Belgium). At an age of one month, only live *Chironomus* larvae were provided every morning (8am) and evening (8pm) in an *ad libitum* quantity to ensure a continuous supply of live food.

### Monitoring life-history traits and activity

Body size was determined at two, 16 and 94 days post-hatching. To this end, individual fish larvae were transferred to a petri dish and photographed from above. Size-calibrated photographs were analyzed using open source image processing software ImageJ 1.50i (Schneider, Rasband & Eliceiri, 2012). Juvenile growth rate was determined as the difference in body size between the age of two and 16 days while adult body size was approximated as the size at an age of 94 days, at which all fish were sexually mature. Juveniles were checked on a daily basis for any signs of male nuptial coloration. The first day at which these signs appeared was used to score the age of sexual maturity in males. Female maturation time was defined and assessed as the first time an egg was deposited.

Starting at an age of 8 weeks—and for a total of 15 spawning events—breeding pairs were assembled for each population. Males and females were paired (novel partner each spawning event) and placed in 1L plastic jars with sterilized sand as a spawning substrate to allow for egg deposition. Excess males or females were paired with a non-experimental fish of the same age and population. After 1 h of spawning, sand was sieved and the amount of eggs was assessed as a measure for female fecundity. Fish were allowed to spawn two to three times per week (Monday, Wednesday and Friday). During the two-week period when activity data was collected (see below), fish were paired only on Monday and Friday.

At an age of 90 days, the activity level of each individual was monitored by a single observer (E Thoré) during three daily sample bursts every Wednesday and Thursday in two consecutive weeks (amounting to a total of 12 sample bursts) (fish: *n* = 121, total observations: *n* = 1,426). Daily sample bursts (Fig. 1D) were organized as follows: morning (start at 8h45), afternoon (start at 12 h 45) and evening (start at 16 h 45), each lasting on average 2 h 30 min, during which individuals were repeatedly scanned for 20 consecutive ‘active or passive’ observations. Fish were scored active and assigned a score of ‘1’ when swimming and passive (score of ‘0’) if they remained motionless during the first three seconds after being spotted—method after Biro & Adriaenssens (2013). Per sample burst, a total activity score was calculated for each individual by taking the sum of the collected scores (maximum score 20, minimum score 0). During the four observation days, tanks were visually separated from each other by means of plastic opaque dividers to prevent social interaction during the activity assays.

All experiments and methods were approved by the ethical committee of KU Leuven (file number: P070/2016).

### Statistical analyses

All statistical analyses were conducted in R 3.3.1 (R Development Core Team, 2008) at a significance level of alpha = 0.05. For all analyses, model assumptions including distributional fit and homogeneity of variances were verified graphically.

Maturation time, juvenile growth, adult body size and peak fecundity (i.e., maximum clutch size per female over the observation period) were analyzed by means of univariate mixed-effect models with Gaussian error distribution using the lme4 package (Bates et al., 2017). Maturation time was analyzed for males and females separately as maturation time was scored differently between sexes. To improve distributional fit, male maturation time was log-transformed; however, we note that the assumption of residual normality could not be entirely met for male maturation, so results should be interpreted with care. Population type (long-lived, short-lived, inbred) and—identity were added to the model as fixed and random effect respectively. For juvenile growth and adult body size, sex and the interaction of sex with population type were added as additional fixed effects. In addition, analyses of maturation time, juvenile growth and peak fecundity were also run with adult body size as covariate.

Fecundity was analyzed using mixed-effects modelling with Poisson distribution (lme4 package). Population type, age and age^2^ (*z*-transformed) were added as fixed effects to the model since fecundity often increases with age in younger animals and decreases again when animals senesce. Fish and population identity were added to the model as random effects. In addition, an observation-level random effect was modelled to accommodate overdispersion.

Significance of the fixed effect was tested using parametric bootstrapping with 1,000 simulations using the afex package (Singmann et al., 2017), and post-hoc differences between population types were assessed by means of Tukey-corrected pairwise comparisons using the lsmeans package (Lenth & Love, 2017). Population-level variation (significance of the random term) was determined by means of log-likelihood ratio tests using the rptR package (Stoffel, Nakagawa & Schielzeth, 2018).

A multilevel random regression approach (lme4 package) with Gaussian error distribution was adopted to analyze activity data and estimate individual behavioral repeatability, following the method outlined by Araya-Ajoy, Mathot & Dingemanse (2015). Activity level was *Z*-transformed and we included a random intercept for population identity, fish identity and the interaction between fish identity and observation day (further on referred to as series), as well as a random slope with respect to moment of day (referring to morning, afternoon and evening, coded as −1, 0 and 1 respectively) at both the fish identity and series hierarchical levels. Covariance between the random intercepts and the slopes of the behavioral reaction norms was allowed. Moment of day (*Z*-transformed), population type and sex—including their full interaction—were added as fixed effects and also the interaction between moment of day and observation day was added. Slope coefficients of activity during the day for each population and both sexes were calculated using the phia package (De Rosario-Martinez & Fox, 2015). Significance of the fixed effects was tested using parametric bootstrapping with 1,000 simulations (afex package), and post-hoc differences were assessed using Tukey-corrected pairwise comparisons (lsmeans package). Repeatability of reaction norm intercepts (i.e., individual average activity level) was calculated as the among-individual variance in intercepts divided by the summation of the among-individual variance in intercepts and the within-individual variance in intercepts among series. Repeatability of reaction norm slopes (i.e., individual diurnal activity change) was calculated as the among-individual variance in slopes divided by the summation of the among-individual variance in slopes and the within-individual variance in slopes among series. See Araya-Ajoy, Mathot & Dingemanse (2015) for a more elaborate discussion on how to calculate repeatability of reaction norm intercepts and slopes. Parametric bootstrapping with 1,000 iterations was used to obtain the 95% confidence interval for the repeatability estimates to determine if repeatability differs significantly from zero (i.e., when 95% CI does not span zero).

Life-history and activity level correlation at the population level was analyzed for both sexes separately by randomly sampling 70% of the original data per population for a total of 1,000 times, each time calculating the pearson-correlation coefficient between maturation time, juvenile growth rate and adult body size (and total fecundity for females) on the one hand and total activity score (summated across the 12 sample bursts) on the other hand. The result was considered significant if the middle 95% of the distribution excluded zero. At the individual level, correlation patterns between life-history and activity level (approximated by total activity score, summated across the 12 sample bursts; i.e., at the unpartitioned phenotypic level) were assessed by means of pearson-correlation tests for both sexes separately. Correlation between life-history and diurnal activity change (at the individual level trait value, i.e., as individual random slopes with respect to moment of day, extracted as best linear unbiased predictors, BLUPs, from the above mixed model) at the population and individual level was analyzed using a similar approach (pearson-correlation tests). It should be noted that using BLUPs is an anti-conservative approach as this method does not carry forward the inherent error around the predictions of individual-level estimates to the subsequent correlation tests (see Houslay & Wilson, 2017).This method, however, allows for a uniform testing of life-history and activity correlations of all traits on both the individual and population level.

## Results

### Life-history

**Figure 2:**
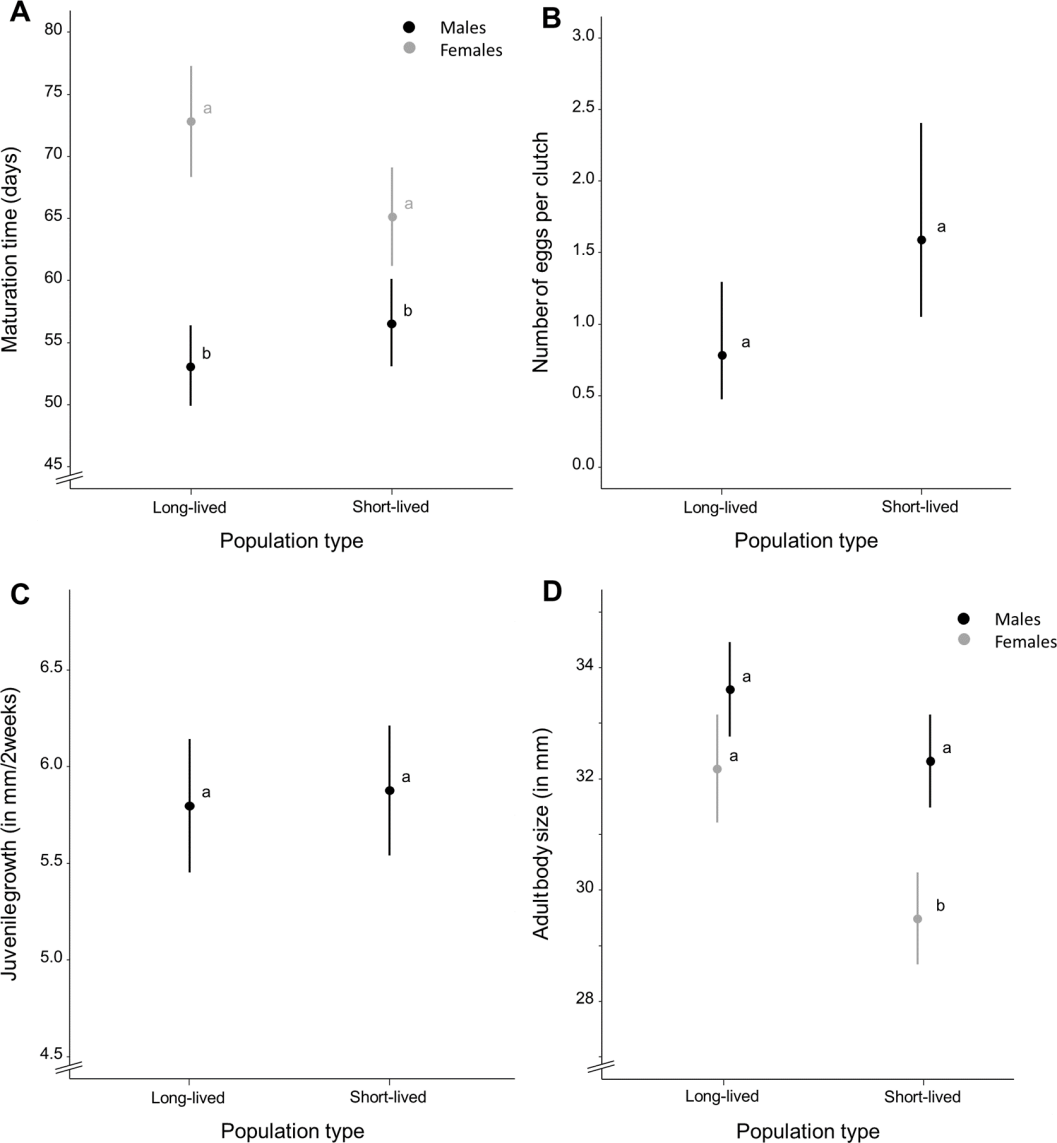
Average life-history trait expression per population type. (A) Maturation time (in days) for each population type and separated by sex. (B) Mean number of eggs per clutch as a measure of female fecundity and (C) mean juvenile growth rate (as the difference in body size between the age of 2 and 16 days, in millimeter) for each population type. (D) Mean adult body size (in millimeter) for each population type, separated by sex. Whiskers delineate the upper and lower 95% confidence limit. Letters indicate significant differences based on Tukey-corrected post-hoc tests.

Wild-derived female fish with short life expectancy reached sexual maturity at mean ± S.E. 65.145 ± 1.927 days whereas females with long life expectancy reached maturity at mean ± S.E. 72.883 ± 2.184 days (Fig. 2A). Females of the homozygous GRZ strain reached maturity at mean ± S.E. 79.428 ± 3.387 days (Fig. S1). Time to reach sexual maturity did not differ significantly between population types for females (*χ*^2^ = 9.273, *p* = 0.106) (Table S1). There was no significant variation for female maturation time within population types (*p* = 0.500). Adding body size as a covariate to this model did not alter these results. Female maturation time was independent of female adult body size (*χ*^2^ =  − 0.351, *p* = 1.000) and did not differ between population types (*χ*^2^ = 9.085, *p* = 0.118).

Wild-derived male fish with short life expectancy reached sexual maturity at mean ± S.E. 56.570 ± 1.031 days, whereas males with long life expectancy reached maturity at mean ± S.E. 53.105 ± 1.030 days (Fig. 2A). Males of the homozygous GRZ strain reached maturity at mean ± S.E. 49.886 ± 1.037 days (Fig. S1). Time to reach sexual maturity did not differ significantly between population types for males (*χ*^2^ = 6.216, *p* = 0.104) (Table S2). There was no significant variation for male maturation time within population types (*p* = 0.500). Adding body size as a covariate to this model did not alter these results. Male maturation time decreased with an increase in male adult body size (*χ*^2^ = 11.703, ***p*** = **0.002**) but did not differ between population types (*χ*^2^ = 7.466, *p* = 0.103).

Female fish with short life expectancy produced mean ± S.E. 1.590 ± 0.337 eggs per clutch, whereas fish with long life expectancy produced mean ± S.E. 0.782 ± 0.201 eggs per clutch (Fig. 2B). Females of the homozygous GRZ strain produced mean ± S.E. 0.344 ± 0.146 eggs per clutch (Fig. S1). There was no overall difference in fecundity between population types (*χ*^2^ = 8.756, *p* = 0.154). There was a significant positive effect of age (*χ*^2^ = 61.616, ***p*** = **0.001**) and age^2^ (*χ*^2^ = 43.397, ***p*** = **0.001**), i.e., a flattening positive relationship between age and fecundity (Table S3). There was significant variation for fecundity within population types (***p*** < **0.001**). Female peak fecundity did not differ between population types (*χ*^2^ = 2.131, *p* = 0.619) (Table S4). Adding body size as a covariate to this model did not alter these results. Peak fecundity was independent of female adult body size (*χ*^2^ = 2.428, *p* = 0.125) and did not differ between population types (*χ*^2^ = 2.628, *p* = 0.535).

Juvenile growth rate (Table S5) did not differ between population types (*χ*^2^ = 4.660, *p* = 0.330) and sexes (*χ*^2^ = 0.035, *p* = 0.860), nor were there sex-specific differences in juvenile growth rate between population types (*χ*^2^ = 0.824, *p* = 0.667) (Fig. 2C). There was no significant variation for juvenile growth rate within population types (*p* = 0.163). Adding body size as a covariate to this model did not alter these results. Juvenile growth rate was positively correlated with adult body size (*χ*^2^ = 5.478, ***p*** = **0.023**) but did not differ between population types (*χ*^2^ = 4.574, *p* = 0.343) and sexes (*χ*^2^ = 1.526, *p* = 0.215). There were no sex-specific differences in juvenile growth rate between population types (*χ*^2^ = 0.194, *p* = 0.913) when adult body size was added as a covariate to the model.

Adult body size (Table S6), averaged over sexes, differed significantly between population types (*χ*^2^ = 12.465, ***p*** = **0.037**) with fish from short-lived ponds (mean ± S.E. 31.097 ± 0.305 mm) being smaller than fish with long life expectancy (mean ± S.E. 32.971 ± 0.322 mm). Fish of the homozygous GRZ strain had a body size of mean ± S.E. 30.569 ± 0.467 mm (Fig. S1). Sexes differed in adult body size (*χ*^2^ = 37.437, ***p*** < **0.001**) with females (mean ± S.E. 30.387 ± 0.277 mm) being smaller than males (mean ± S.E. 32.720 ± 0.245 mm). Adult body size differed similarly between population types for both sexes (*χ*^2^ = 4.223, *p* = 0.119); however, post-hoc analyses revealed significant differences in body size between population types for females whereas this was only a trend for males (Fig. 2D). There was no significant variation for adult body size within population types (*p* = 1.000).

### Behavior

**Figure 3:**
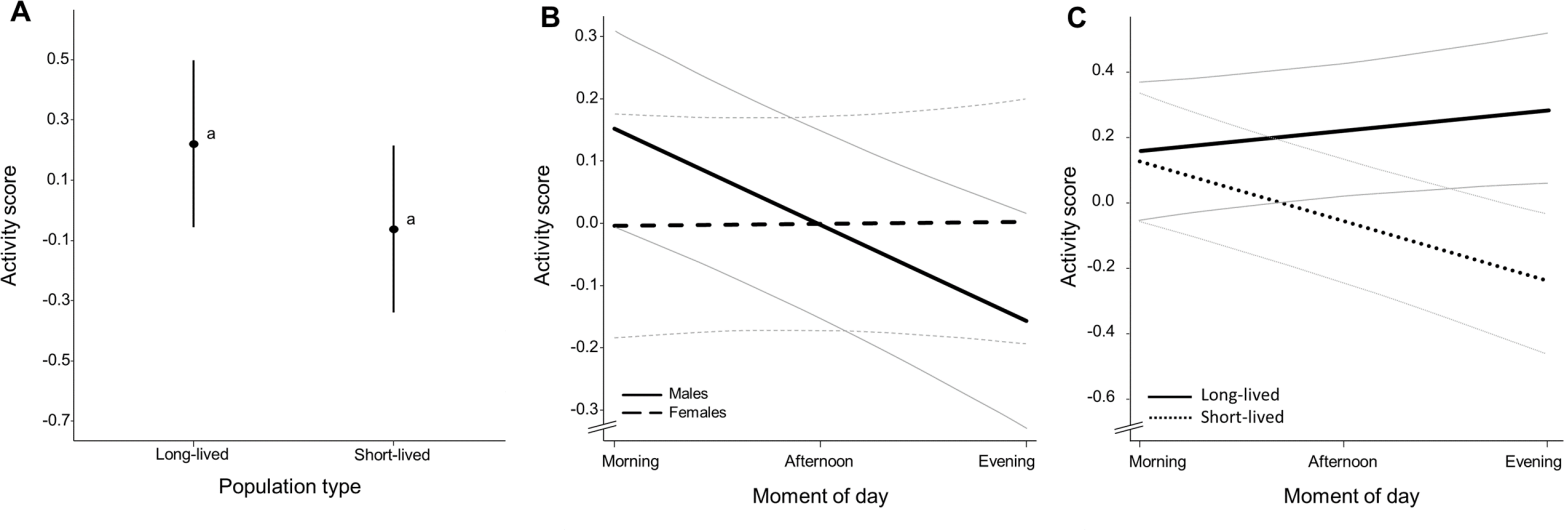
Average activity score (*Z*-transformed scores). (A) Mean activity score for each population type. Whiskers delineate the upper and lower 95% confidence limit. Letters indicate significant differences based on Tukey-corrected post-hoc tests. (B) Average change in activity score over the course of day for males and females (including 95% confidence bands outlined in grey) and (C) for each population type. Slope coefficients and corresponding *p*-values are given in Table 2.

Activity level (Table S7) did not differ significantly between population types (*χ*^2^ = 7.750, *p* = 0.078, Fig. 3A), sexes (*χ*^2^ = 0. 060, *p* = 0.789) and observation days (*χ*^2^ = 7.447, *p* = 0.065) and there were no sex-dependent differences in activity level between population types (*χ*^2^ = 0.363, *p* = 0.832). Activity level differed significantly over the course of day (*χ*^2^ = 7.418, ***p*** = **0.016**). Diurnal changes in activity were dependent on observation day (*χ*^2^ = 27.076, ***p*** = **0.001**) and sex (*χ*^2^ = 7.746, ***p*** = **0.003**) with males exhibiting a decline in activity towards the evening while this change in activity level was not apparent for females (Fig. 3B, slope coefficients represented in Table 2, see Fig. S2 for inclusion of raw data). Population types differed in their diurnal activity pattern (*χ*^2^ = 18.063, ***p*** = **0.001**) with fish from short-lived ponds exhibiting a decline in activity towards the evening while this activity change was not apparent in fish with long life expectancy (Fig. 3C, slope coefficients represented in Table 2, see Fig. S3 for inclusion of raw data). Fish of the homozygous GRZ strain also exhibit a decline in activity towards the evening (Fig. S4). There were no sex-dependent differences in diurnal activity change between population types (*χ*^2^ = 0.081, *p* = 0.957). Slope coefficients (of activity during the day) per sex for each population are represented in Table S8. Individual average activity level was significantly repeatable with *R*_intercept_ = 0.550 (95% CI [0.426–0.658]). Reaction norm slopes as a measure for individual diurnal activity change were significantly repeatable with *R*_slope_ = 0.731 (95% CI [0.393–0.999]) (see Fig. S5 for a representation of individual level reaction norms).

**Table 2:** Slope coefficient of activity throughout the day for each population type and both sexes.

		Value	Df	*χ*^2^	*p*-value
Population	Long-lived	0.071	1	2.719	0.099
	Short-lived	**−0.172**	1	19.498	<0.001^*^
Sex	F	0.002	1	0.001	0.970
	M	**−0.156**	1	20.202	<0.001^*^

**Notes.**

Values that are significantly different from zero are shown in bold.

* *p*-values < 0.05 are indicated with an asterisk (*).

### Life-history and activity correlation

Overall, life-history traits and activity (including diurnal activity change) were not correlated at the population or individual level (Table 3). However, female adult body size was positively correlated (*r* = 0.314, *n* = 51, *p* = 0.025) with activity on the individual level.

**Table 3:** Pearson correlation coefficient (*r*) between life-history traits and total activity score and between life-history traits and diurnal activity change on the individual and population level for both sexes.

	Life-history trait	*r*_activity_ (95% confidence interval)	*r*_diurnal change_ (95% confidence interval)
*Individual level*			
Males	Maturation time	0.094 (−0.151; 0.328)	0.107 (−0.138; 0.340)
	Juvenile growth rate	0.238 (−0.003; 0.452)	−0.097 (−0.329; 0.147)
	Adult body size	0.209 (−0.033; 0.428)	−0.203 (−0.422; 0.039)
Females	Maturation time	−0.059 (−0.324; 0.214)	0.145 (−0.130; 0.400)
	Juvenile growth rate	−0.109 (−0.371; 0.169)	−0.048 (−0.316; 0.228)
	Adult body size	**0.314** (0.042; 0.543)*	0.003 (−0.272; 0.279)
	Fecundity	0.082 (−0.193; 0.344)	−0.119 (−0.378; 0.156)
*Population level*			
Males	Maturation time	−0.268 (−0.872; 0.397)	−0.130 (−0.868; 0.803)
	Juvenile growth rate	0.520 (−0.229; 0.953)	−0.172 (−0.905; 0.628)
	Adult body size	0.502 (−0.226; 0.936)	−0.130 (−0.857; 0.610)
Females	Maturation time	−0.229 (−0.878; 0.408)	−0.132 (−0.885; 0.803)
	Juvenile growth rate	0.536 (−0.188; 0.962)	−0.147 (−0.889; 0.686)
	Adult body size	0.524 (−0.322; 0.936)	−0.121 (−0.827; 0.639)
	Fecundity	−0.230 (−0.855; 0.623)	0.025 (−0.843; 0.913)

**Notes.**

Significant values are shown in bold; 95% confidence intervals that exclude zero are indicated with an asterisk (*). For population-level correlations, the middle 95% distribution interval of bootstrapped correlations is given.

## Discussion

Our results demonstrate sex-specific variation in adult body size and diurnal activity change among populations that originate from ponds that differ in permanence. Overall, our results did not demonstrate a relationship between life-history and behavior at any level of biological organization as postulated by the POLS. In addition, there was no pond permanence-dependent divergence in maturation time, juvenile growth rate, fecundity and average activity level. Variation at the population-level is assumed to be genetically underpinned, as populations differ even after at least three generations of breeding under common garden laboratory conditions. Furthermore, the fact that individuals differed consistently in locomotor activity and diurnal activity change is reflective of personality variation and consistent inter-individual variation in behavioral patterns respectively.

With respect to life-history, *N. furzeri* fish with short life expectancy (short-lived ponds) differed in adult body size from fish with long life expectancy (long-lived ponds) and there was a trend towards faster female maturation for shorter-lived fish. Faster maturation of populations from short-lived ponds (arid region) allows individuals to produce the next generation of drought resistant eggs before early drying of ponds and earlier than populations from longer-lived ponds (wet region). Especially in short-lived ponds, demographic modelling illustrates that early maturation can be an important determinant of long term population growth rates for organisms with a life cycle similar to that of *N. furzeri* (Pinceel et al., 2016). In addition, life-history theory predicts a higher maturation rate to be offset by a reduced adult body size (Hollis, Keller & Kawecki, 2016). Although there was a trend for faster maturation of females from short-lived ponds and females from short-lived ponds were smaller as adults than females from long-lived ponds, an opposite association was found for male maturation time and males from different population types did not differ in adult body size. These sex-associated differences illustrate the importance of including both sexes when studying life-history responses. Whereas the negative association between female maturation rate and adult body size is broadly supported in literature, this is far less so for males (Hollis, Keller & Kawecki, 2016). Likely, there are additional fitness advantages associated with early male maturation, such as accommodating aggressive male competition for mates (Reichard, Polačik & Sedláček, 2009) that drive selection for early maturation. Should competition for mating opportunities be stronger in long-lived ponds due to, for instance, the occasional occurrence of multiple age cohorts within the same pond (Reichard et al., 2017) then early maturation could entail a reproductive advantage (Hollis, Keller & Kawecki, 2016). Alternatively and non-mutually exclusive, maturation time might be correlated with traits that were not assessed in the current study, such as sex-specific immunological traits, that may underlie sex-related differences in maturation time.

Consistent with an earlier study by Blažek et al. (2016), our results only partly support associations between pond permanence and life-history trait variation. While fish from short- and long-lived populations differed in adult body size, no such differences in juvenile growth rate and fecundity emerged. Possibly, fish from these habitats are already at their physiological limit and developmental constraints prevent individuals from short-lived pools to invest even more energy in rapid somatic growth and higher reproductive output (Blažek et al., 2016; Grégoir et al., 2018). If a trade-off between investing in current versus future reproduction exists, then the POLS predicts variation in life-history to co-evolve with behavioral traits that mediate life-history traits involved in this trade-off (Réale et al., 2010; Dammhahn et al., 2018). However, in addition to lack of differences in growth rate and fecundity, our results did not demonstrate divergence in average activity level that can be associated with pond permanence. Also, the average activity level did not differ between sexes, which contradicts the prediction that males and females are positioned at the fast and slow end of the pace-of-life continuum, respectively (Debecker et al., 2016). Furthermore, our results do not confirm a link between activity level and life-history traits. This implies that fish from different population types and sexes can accelerate maturation in the absence of activity changes under *ad libitum* feeding conditions. While a positive correlation between activity level and adult female body size was found on the individual level, this result may represent a false positive given that a large number of correlation tests were conducted (Type I error). It should be noted that, because correlation analyses with BLUPs are anti-conservative, we cannot exclude that some relationships are not detected using this approach. Therefore, future research could aim for test designs that are adapted to the use of a multivariate mixed modelling approach as a data hungry but more powerful approach (Houslay & Wilson, 2017).

Unlike average activity level, our results show pond permanence-dependent divergence in diurnal activity change. Fish originating from short-lived ponds with a short life expectancy exhibit a decline in activity towards the evening. Such a diurnal activity pattern was absent in fish from long-lived ponds and, hence, the presence of this diurnal pattern coincides with pond permanence and life expectancy. Populations with a fast pace-of-life are expected to exhibit a higher metabolic rate than slow-paced populations (Réale et al., 2010). Since individuals with a high metabolic rate are confronted with higher levels of oxidative damage due to an increased production of reactive oxygen species, the decrease in activity throughout the day may reflect a homeostatic need (Larcombe et al., 2010; Stuber et al., 2015). Inactivity or sleep was, for instance, demonstrated to have a restorative function in mice (Xie et al., 2013). Clear life-history divergence among populations from ponds that differ in permanence could, however, not be confirmed in this study. Therefore, we cannot conclude that a fast pace-of-life underlies diurnal activity patterns in *Nothobranchius* killifish. Future research should complement the behavioral analyses with physiological data to further explore this hypothesis.

In addition to divergence in diurnal activity change with respect to pond permanence, our results also demonstrate sex-specific differences in diurnal activity pattern with males becoming less active towards the evening while females maintain the same activity level throughout the day. Daily arrhythmicity, or around-the-clock activity, has been described in a range of species. Next to differences in metabolic rate as a potential explanation for this variation, several hypotheses have been framed to explain daily arrhythmicity (Bloch et al., 2013). Among these, reproductive behavior has been described as a potential determinant of daily arrhythmicity. For instance, reproductive queens of different hymenopteran species are active around the clock to maximize their fecundity (Johnson et al., 2010; Bloch et al., 2013). Sex-dependent differences in sleep and sleep-like behavior have also been illustrated in bird species including great tits (*Parus major*) (Stuber et al., 2015) and blue tits (*Cyanistes caeruleus*) (Steinmeyer et al., 2010) and could be the result of sexual selection. Although activity was not monitored at night and only at select points during the day in the current study, the apparent absence of a decline in activity over the course of the day in females might be an adaptive strategy to maximize reproductive output by being available for reproduction throughout the whole day. However, if this is the case, a similar pattern would be expected for males. For instance, males of the polygynous pectoral sandpiper (*Calidris melanotos*) have been shown to increase their activity to increase the amount of sired offspring (Lesku et al., 2012). Such a strategy could, however, not be confirmed in the current study. Since *N. furzeri* males are highly conspicuous to visual predators due to their bright colors, the observed decline in activity may be an adaptation to minimize the risk of avian predation. Wild populations of *N. furzeri* are reported to be strongly female biased, which could be caused by preferential bird predation on males (Reichard, Polačik & Sedláček, 2009). Adaptive inactivity might, therefore, represent an energy-conserving strategy when the costs of being active exceed the benefits (Siegel, 2009). Whereas *Nothobranchius* killifish exhibit stable locomotor rhythms and are diurnal (i.e., they exhibit nocturnal periods of inactivity) (Lucas-Sánchez et al., 2013; Lucas-Sánchez et al., 2014), future research should aim to assess day-round (24 h) patterns of activity to get a more complete image of daily activity changes.

Behavioral patterns like diurnal activity change have recently been suggested as overlooked dimensions of within-species behavioral diversity and circadian behavioral variation has been argued to be a novel axis of animal personality (Dingemanse et al., 2010; Randler, 2014; Alós, Martorell-Barceló & Campos-Candela, 2017). Describing natural variation in diurnal activity patterns is an important step in attempting to elucidate its ecological function and evolutionary architecture (Lima et al., 2005). Our results demonstrate repeatable inter-individual differences in activity and diurnal activity change for the killifish *N. furzeri*. In human psychology, variation in diurnal activity patterns has often been associated with variation in personality and life style (Adan et al., 2012). While variation in diurnal activity change is of interest in various sectors (clinic, academic, labour) (Adan et al., 2012), it remains understudied in ecology and evolutionary biology (Helm & Visser, 2010). How variation in diurnal activity change relates to variation in traditional personality traits such as boldness and exploration behavior in animals and its integration in the POLS framework opens an interesting avenue for research and should be subject to further investigation (Randler, 2014; Stuber et al., 2015). For instance, Tudorache et al. (2018) demonstrated that circadian rhythmicity at the molecular, endocrine and behavioral level was strongly associated with risk-taking behavior as a proxy for coping style in zebrafish (*Danio rerio*). Proactive fish were shown to display a robust rhythm with a large amplitude, whereas circadian rhythmicity was largely lacking in reactive fish. As argued by the authors of the study, this finding suggests that circadian rhythmicity could be an integral part of individual life-history strategies. Only four studies, three on birds (Steinmeyer et al., 2010; Stuber et al., 2014; Stuber et al., 2015) and one on fish (Alós, Martorell-Barceló & Campos-Candela, 2017), assessed repeatability as a measure for the consistency of between-individual differences in diurnal activity change. Our study delivers an effective ‘proof of principle’ for the existence of repeatable inter-individual variation in average activity levels and diurnal activity change in *N. furzeri*, which could be reflective of personality variation. Although we could not show correlation between diurnal activity pattern and life-history, diurnal activity change differed between sexes and between fish with different life expectancy along a pond permanence gradient. Nevertheless, given these results and given that diurnal activity change could potentially be a system- or species-specific integrated component of POLS, we argue that it would be interesting for future studies to include activity patterns as these could further add to the realism of pace-of-life studies. If that is the case, only a complete documentation of where such correlations occur or do not occur will give sufficient insight into the underlying mechanism that determines the occurrence of such correlations. In addition, we note that, given that only four natural populations could be used in this study, it would be interesting for future studies to include more populations along the pond permanence gradient in the field to increase data resolution and to add more realism to the experimental setup, while relating life-history and behavioral expression to direct measurements of pond permanence.

## Conclusions

In conclusion, our results demonstrate individual, sex and population dependent variation in certain life-history and behavioral traits in *N. furzeri*, supporting multi-level approaches to elucidate the ecology and evolution that underpin phenotypic diversity. The absence of an association between activity level and life-history aspects of pace-of-life on the individual level is inconsistent with the POLS framework. On the population level, however, the POLS framework does not seem to apply given that no differences in life-history strategies between populations could be confirmed. Despite a large number of studies within the POLS framework, many failed to identify a stable correlation between activity and life-history, which causes scientists to question its generality. Consequently, an evaluation and refinement of the framework is essential (Adriaenssens, 2017; Dammhahn et al., 2018). Unlike average activity level, diurnal activity change was associated with pond permanence and sex. Furthermore, our findings support that consistent inter-individual differences in diurnal activity pattern might reflect a long overlooked dimension of animal personality. Although correlation between diurnal activity pattern and life-history was not confirmed in this study, we argue that trait covariation could be species specific and future efforts should examine if diurnal activity change could be integrated in the POLS framework in other systems to more closely approximate real-life situations and better understand behavioral diversity and its fitness consequences.

##  Supplemental Information

10.7717/peerj.7177/supp-1 Table S1 The results from the linear mixed effects model for female maturation time

10.7717/peerj.7177/supp-2 Table S2 The results from the linear mixed effects model for male maturation time

10.7717/peerj.7177/supp-3 Table S3 The results from the linear mixed effects model for fecundity Note: *p*-values < 0.05 are indicated with an asterisk (*).

10.7717/peerj.7177/supp-4 Table S4 The results from the linear mixed effects model for peak fecundity Note: *p*-values < 0.05 are indicated with an asterisk (*).

10.7717/peerj.7177/supp-5 Table S5 The results from the linear mixed effects model for juvenile growth Note: *p*-values < 0.05 are indicated with an asterisk (*).

10.7717/peerj.7177/supp-6 Table S6 The results from the linear mixed effects model for adult body size Note: *p*-values < 0.05 are indicated with an asterisk (*).

10.7717/peerj.7177/supp-7 Table S7 The results from the linear mixed effects model for activity data Note: *p*-values < 0.05 are indicated with an asterisk (*).

10.7717/peerj.7177/supp-8 Table S8 Slope coefficient of activity throughout the day for each population type per sex Note: Values that are significantly different from zero are shown in bold; *p*-values < 0.05 are indicated with an asterisk (*).

10.7717/peerj.7177/supp-9 Figure S1 Average life-history trait expression per population type (inbred GRZ strain included) (A) Maturation time (in days) for each population type and separated by sex. (B) Mean number of eggs per clutch as a measure of female fecundity and (C) mean juvenile growth rate (as the difference in body size between the age of 2 and 16 days, in millimetre) for each population type. (D) Mean adult body size (in millimetre) for each population type, separated by sex. Whiskers delineate the upper and lower 95% confidence limit. Letters indicate significant differences based on Tukey-corrected post-hoc tests.

10.7717/peerj.7177/supp-10 Figure S2 Average activity score (Z-transformed scores) Average change in activity score over the course of day for males and females (including 95% confidence bands outlined with dashed lines), including raw data points. Males are indicated in blue, females are indicated in red.

10.7717/peerj.7177/supp-11 Figure S3 Average activity score (*Z*-transformed scores) Average change in activity score over the course of day for each population type (including 95% confidence bands outlined with dashed lines), including raw data points. Long-lived population type is indicated in green, short-lived population type is indicated in blue and the short-lived GRZ population is indicated in red.

10.7717/peerj.7177/supp-12 Figure S4 Average activity score (*Z*-transformed scores) (A) Mean activity score for each population type (GRZ strain included). Whiskers delineate the upper and lower 95% confidence limit. Letters indicate significant differences based on Tukey-corrected post-hoc tests. (B) Average change in activity score over the course of day for males and females (including 95% confidence bands outlined in grey) and (C) for each population type. Slope coefficients and corresponding p-values are given in Table 2 for the long-lived and short-lived population types. Slope value of activity throughout the day for the GRZ strain is −0.131 (*χ*^2^ = 4.247, *p* = 0.039).

10.7717/peerj.7177/supp-13 Figure S5 Individual level reaction norms of activity score (*Z*-transformed scores) over the course of day For clarity only 30 random individuals are shown.

10.7717/peerj.7177/supp-14 Dataset S1 Activity data

10.7717/peerj.7177/supp-15 Dataset S2 Life-history data

10.7717/peerj.7177/supp-16 Dataset S3 Fecundity data

## Additional Information and Declarations

### Data Availability

The following information was supplied regarding data availability: Data are available in the Supplemental Files.
