# Supplementary material for: Population-, sex- and individual level divergence in life-history and activity patterns in an annual killifish
Source: PeerJ. 2019 Jun 27;7:e7177. doi: 10.7717/peerj.7177 (PMC6599669; doi:10.7717/peerj.7177)
Supplement: Table S1 [file peerj-07-7177-s001.docx]

**Table S1**: The results from the linear mixed effects model for female maturation time.

| *Fixed effects* | *Estimate* | *Standard Error* | *t value* |
| --- | --- | --- | --- |
| (Intercept) | 72.486 | 1.489 | 48.680 |
| Type1 | 6.943 | 2.458 | 2.820 |
| Type2 | 0.397 | 1.951 | 0.200 |
| *Random effects* | *Name* | *Variance* | *Standard dev.* |
| Population | (Intercept) | 3.033 | 1.742 |
| Residual |  | 59.075 | 7.686 |
|  |  |  |  |
| Number of observations: 53 | | | |
| Groups: Population, 5 | | | |
