# Supplementary material for: Population-, sex- and individual level divergence in life-history and activity patterns in an annual killifish
Source: PeerJ. 2019 Jun 27;7:e7177. doi: 10.7717/peerj.7177 (PMC6599669; doi:10.7717/peerj.7177)
Supplement: Table S2 [file peerj-07-7177-s002.docx]

**Table S2**: The results from the linear mixed effects model for male maturation time.

| *Fixed effects* | *Estimate* | *Standard Error* | *t value* |
| --- | --- | --- | --- |
| (Intercept) | 3.972 | 0.019 | 221.400 |
| Type1 | -0.063 | 0.028 | -2.210 |
| Type2 | < -0.001 | 0.025 | -0.010 |
| *Random effects* | *Name* | *Variance* | *Standard dev.* |
| Population | (Intercept) | < 0.001 | < 0.001 |
| Residual |  | 0.023 | 0.152 |
|  |  |  |  |
| Number of observations: 68 | | | |
| Groups: Population, 5 | | | |
