# Supplementary material for: Population-, sex- and individual level divergence in life-history and activity patterns in an annual killifish
Source: PeerJ. 2019 Jun 27;7:e7177. doi: 10.7717/peerj.7177 (PMC6599669; doi:10.7717/peerj.7177)
Supplement: Table S3 — Note: p-values < 0.05 are indicated with an asterisk (*). [file peerj-07-7177-s003.docx]

**Table S3**: The results from the linear mixed effects model for fecundity.

| *Fixed effects* | *Estimate* | *Standard Error* | *z value* | *Pr(>\|z\|)* |
| --- | --- | --- | --- | --- |
| (Intercept) | -0.897 | 0.191 | -4.688 | < 0.001* |
| Type1 | -0.785 | 0.292 | -2.690 | 0.007* |
| Type2 | 0.038 | 0.219 | 0.173 | 0.863 |
| Age | 9.240 | 1.215 | 7.603 | < 0.001* |
| Age² | -7.553 | 1.173 | -6.441 | < 0.001* |
|  |  |  |  |  |
| *Random effects* | *Name* | *Variance* | *Standard dev.* |  |
| Observation | (Intercept) | 2.490 | 1.578 |  |
| Individual | (Intercept) | 0.760 | 0.872 |  |
| Population | (Intercept) | < 0.001 | < 0.001 |  |
|  |  |  |  |  |
| Number of observations: 810 | | | |  |
| Groups: Observation, 810; Individual, 54; Population, 5 | | | |  |

Note: p-values < 0.05 are indicated with an asterisk (*).
