# Supplementary material for: Population-, sex- and individual level divergence in life-history and activity patterns in an annual killifish
Source: PeerJ. 2019 Jun 27;7:e7177. doi: 10.7717/peerj.7177 (PMC6599669; doi:10.7717/peerj.7177)
Supplement: Table S4 — Note: p-values < 0.05 are indicated with an asterisk (*). [file peerj-07-7177-s004.docx]

**Table S4**: The results from the linear mixed effects model for peak fecundity.

| *Fixed effects* | *Estimate* | *Standard Error* | *df* | *t value* | *Pr(>\|t\|)* |
| --- | --- | --- | --- | --- | --- |
| (Intercept) | 21.539 | 3.316 | 6.071 | 6.495 | 0.001* |
| Type1 | -3.539 | 5.344 | 6.571 | -0.662 | 0.530 |
| Type2 | -3.225 | 4.380 | 5.976 | -0.736 | 0.489 |
| *Random effects* | *Name* | *Variance* | *Standard dev.* |  |  |
| Population | (Intercept) | 33.350 | 5.775 |  |  |
| Residual |  | 135.370 | 11.635 |  |  |
|  |  |  |  |  |  |
| Number of observations: 53 | | | |  |  |
| Groups: Population, 5 | | | |  |  |

Note: p-values < 0.05 are indicated with an asterisk (*).
