# Supplementary material for: Population-, sex- and individual level divergence in life-history and activity patterns in an annual killifish
Source: PeerJ. 2019 Jun 27;7:e7177. doi: 10.7717/peerj.7177 (PMC6599669; doi:10.7717/peerj.7177)
Supplement: Table S5 — Note: p-values < 0.05 are indicated with an asterisk (*). [file peerj-07-7177-s005.docx]

**Table S5**: The results from the linear mixed effects model for juvenile growth.

| *Fixed effects* | *Estimate* | *Standard Error* | *df* | *t value* | *Pr(>\|t\|)* |
| --- | --- | --- | --- | --- | --- |
| (Intercept) | 5.689 | 0.069 | 5.640 | 82.880 | < 0.001* |
| Type1 | -0.296 | 0.111 | 6.280 | -2.658 | 0.036* |
| Type2 | 0.109 | 0.090 | 5.400 | 1.209 | 0.277 |
| Sex1 | -0.010 | 0.053 | 114.390 | -0.189 | 0.850 |
| Type1:Sex1 | -0.021 | 0.087 | 113.970 | -0.239 | 0.812 |
| Type2:Sex1 | 0.056 | 0.068 | 115.190 | 0.818 | 0.415 |
| *Random effects* | *Name* | *Variance* | *Standard dev.* |  |  |
| Population | (Intercept) | 0.009 | 0.093 |  |  |
| Residual |  | 0.245 | 0.500 |  |  |
|  |  |  |  |  |  |
| Number of observations: 119 | | | |  |  |
| Groups: Population, 5 | | | |  |  |

Note: p-values < 0.05 are indicated with an asterisk (*).
