# Supplementary material for: Population-, sex- and individual level divergence in life-history and activity patterns in an annual killifish
Source: PeerJ. 2019 Jun 27;7:e7177. doi: 10.7717/peerj.7177 (PMC6599669; doi:10.7717/peerj.7177)
Supplement: Table S6 — Note: p-values < 0.05 are indicated with an asterisk (*). [file peerj-07-7177-s006.docx]

**Table S6**: The results from the linear mixed effects model for adult body size.

| *Fixed effects* | *Estimate* | *Standard Error* | *df* | *t value* | *Pr(>\|t\|)* |
| --- | --- | --- | --- | --- | --- |
| (Intercept) | 31.457 | 0.178 | 118 | 176.726 | < 0.001* |
| Type1 | -0.888 | 0.288 | 118 | -3.089 | 0.003* |
| Type2 | 1.440 | 0.236 | 118 | 6.093 | < 0.001* |
| Sex1 | -1.182 | 0.178 | 118 | -6.638 | < 0.001* |
| Type1:Sex1 | -0.235 | 0.288 | 118 | -0.818 | 0.415* |
| Type2:Sex1 | 0.470 | 0.236 | 118 | 1.988 | 0.049* |
| *Random effects* | *Name* | *Variance* | *Standard dev.* |  |  |
| Population | (Intercept) | < 0.001 | < 0.001 |  |  |
| Residual |  | 3.036 | 1.742 |  |  |
|  |  |  |  |  |  |
| Number of observations: 118 | | | |  |  |
| Groups: Population, 5 | | | |  |  |

Note: p-values < 0.05 are indicated with an asterisk (*).
