# Supplementary material for: Population-, sex- and individual level divergence in life-history and activity patterns in an annual killifish
Source: PeerJ. 2019 Jun 27;7:e7177. doi: 10.7717/peerj.7177 (PMC6599669; doi:10.7717/peerj.7177)
Supplement: Table S7 — Note: p-values < 0.05 are indicated with an asterisk (*). [file peerj-07-7177-s007.docx]

**Table S7**: The results from the linear mixed effects model for activity data.

| *Fixed effects* | *Estimate* | *Standard Error* | *df* | *t value* | *Pr(>\|t\|)* | |  |
| --- | --- | --- | --- | --- | --- | --- | --- |
| (Intercept) | -0.039 | 0.060 | 118 | -0.663 | 0.508 | |  |
| Moment of day | -0.077 | 0.028 | 122 | -2.767 | 0.007* | |  |
| Observation day 1 | 0.088 | 0.047 | 351 | 1.878 | 0.061 | |  |
| Observation day 2 | 0.049 | 0.047 | 351 | 1.053 | 0.293 | |  |
| Observation day 3 | -0.106 | 0.047 | 352 | -2.245 | 0.025* | |  |
| Type 1 | -0.238 | 0.097 | 118 | -2.464 | 0.015* | |  |
| Type 2 | 0.261 | 0.079 | 118 | 3.314 | 0.001* | |  |
| Sex 1 | 0.015 | 0.060 | 118 | 0.245 | 0.807 | |  |
| Moment of day : Observation day 1 | 0.111 | 0.030 | 357 | 3.656 | < 0.001* | |  |
| Moment of day : Observation day 2 | -0.141 | 0.030 | 357 | -4.652 | < 0.001* | |  |
| Moment of day : Observation day 3 | 0.044 | 0.031 | 358 | 1.431 | 0.153 | |  |
| Moment of day : Type 1 | -0.053 | 0.045 | 123 | -1.179 | 0.241 | |  |
| Moment of day : Type 2 | 0.148 | 0.037 | 123 | 4.006 | < 0.001* | |  |
| Moment of day : Sex 1 | 0.079 | 0.028 | 122 | 2.827 | 0.005* | |  |
| Type 1 : Sex 1 | 0.058 | 0.097 | 118 | 0.603 | 0.547 | |  |
| Type 2 : Sex 1 | -0.031 | 0.079 | 118 | -0.390 | 0.697 | |  |
| Moment of day : Type 1 : Sex 1 | 0.004 | 0.045 | 123 | 0.086 | 0.932 | |  |
| Moment of day : Type 2 : Sex 1 | -0.010 | 0.037 | 123 | -0.263 | 0.793 | |  |
| *Random effects* | *Name* | *Variance* | *Standard dev.* | *Correlation* |  | |  |
| Individual : Observation day | (Intercept) | 0.214 | 0.463 |  |  | |  |
| Moment of day |  | 0.014 | 0.120 | 0.130 |  | |  |
| Individual | (Intercept) | 0.255 | 0.505 |  |  | |  |
| Moment of day |  | 0.037 | 0.193 | 0.190 |  | |  |
| Population | (Intercept) | < 0.001 | < 0.001 |  |  | |  |
| Residual |  | 0.402 | 0.434 |  |  | |  |
|  |  |  |  |  |  | |  |
| Number of observations: 1426 | | | | | |  | |
| Groups: Individual : Observation day, 476; Individual, 121; Population, 5 | | | | | |  | |

Note: p-values < 0.05 are indicated with an asterisk (*).
