# Supplementary material for: Population-, sex- and individual level divergence in life-history and activity patterns in an annual killifish
Source: PeerJ. 2019 Jun 27;7:e7177. doi: 10.7717/peerj.7177 (PMC6599669; doi:10.7717/peerj.7177)
Supplement: Table S8 — Note: Values that are significantly different from zero are shown in bold; p-values < 0.05 are indicated with an asterisk (*). [file peerj-07-7177-s008.docx]

**Table S8**: Slope coefficient of activity throughout the day for each population type per sex.

| Population | Sex | Sample size | Value | Df | χ² | p-value |
| --- | --- | --- | --- | --- | --- | --- |
| Inbred | F | 7 | -0.048 | 1 | 0.200 | 0.655 |
| long-lived | F | 19 | **0.140** | 1 | 4.581 | 0.032* |
| short-lived | F | 27 | -0.087 | 1 | 2.604 | 0.107 |
| Inbred | M | 17 | **-0.213** | 1 | 9.856 | 0.002* |
| long-lived | M | 26 | 0.001 | 1 | < 0.001 | 0.977 |
| short-lived | M | 25 | **-0.256** | 1 | 20.932 | < 0.001* |

Note: Values that are significantly different from zero are shown in bold; p-values < 0.05 are indicated with an asterisk (*).
