# Supplementary figures and images for: Population-, sex- and individual level divergence in life-history and activity patterns in an annual killifish
Source: PeerJ. 2019 Jun 27;7:e7177. doi: 10.7717/peerj.7177 (PMC6599669; doi:10.7717/peerj.7177)

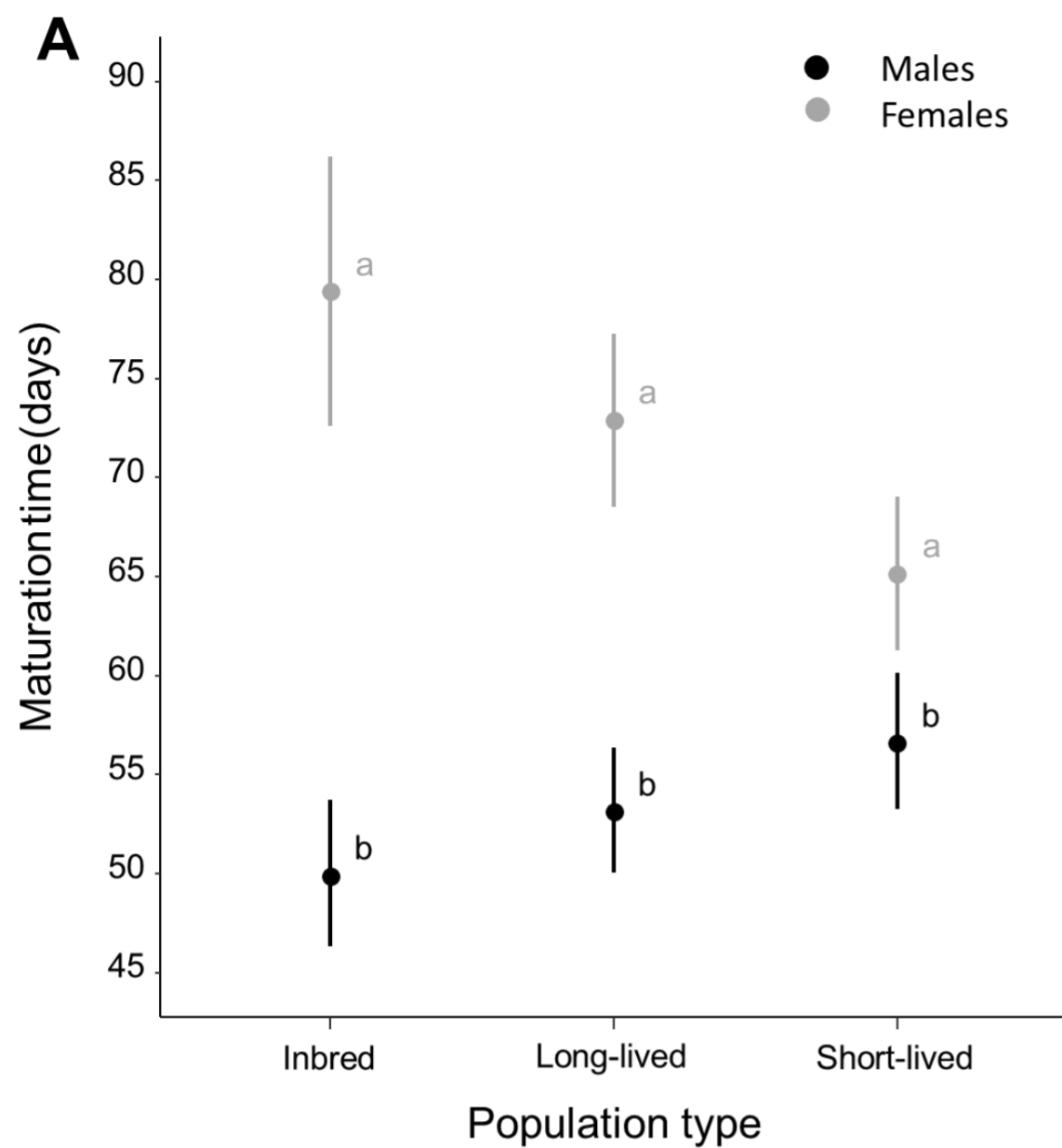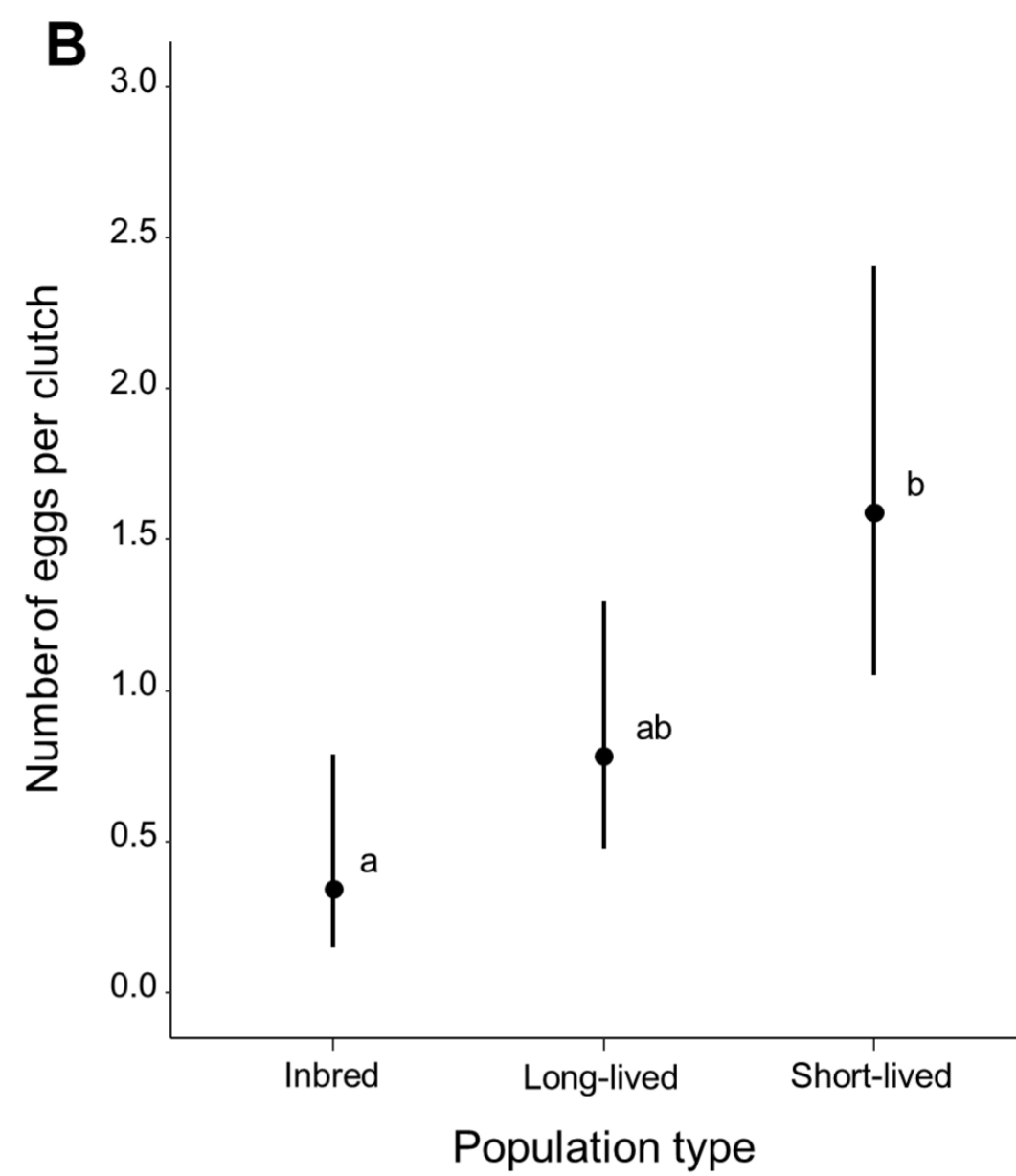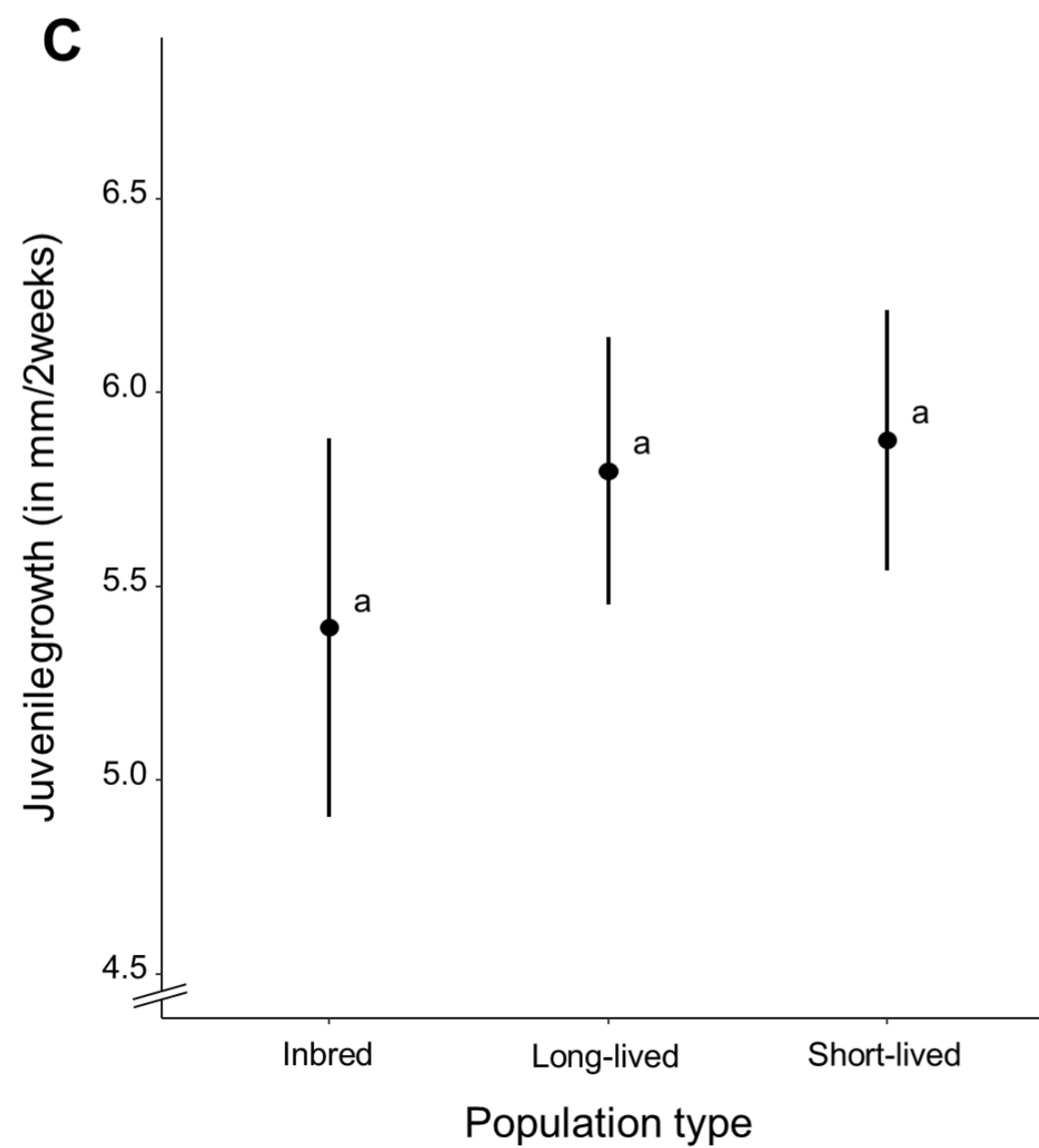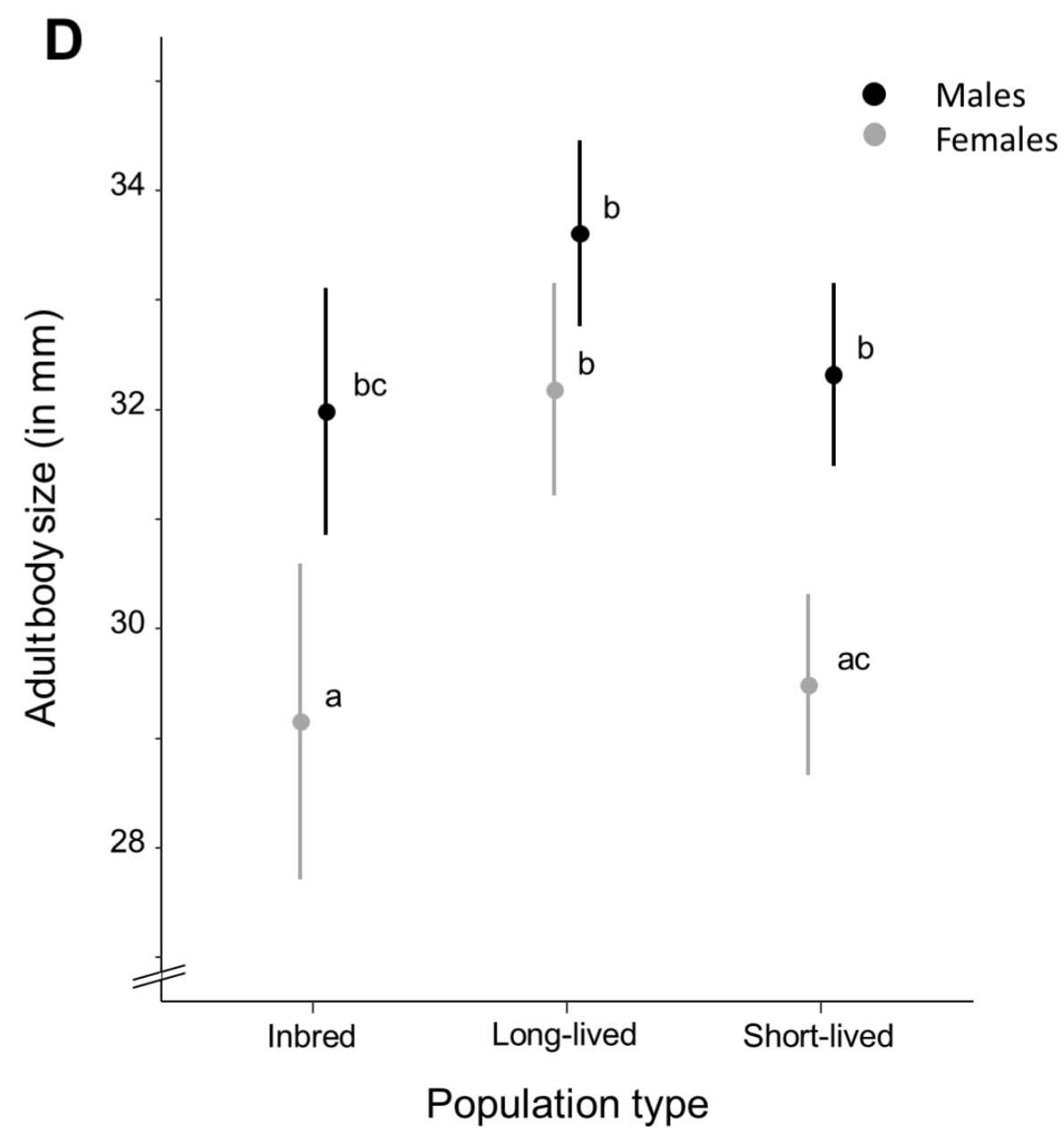

Supplement: Figure S1 — (A) Maturation time (in days) for each population type and separated by sex. (B) Mean number of eggs per clutch as a measure of female fecundity and (C) mean juvenile growth rate (as the difference in body size between the age of 2 and 16 days, in millimetre) for each population type. (D) Mean adult body size (in millimetre) for each population type, separated by sex. Whiskers delineate the upper and lower 95% confidence limit. Letters indicate significant differences based on Tukey-corrected post-hoc tests. [file peerj-07-7177-s009.pdf]

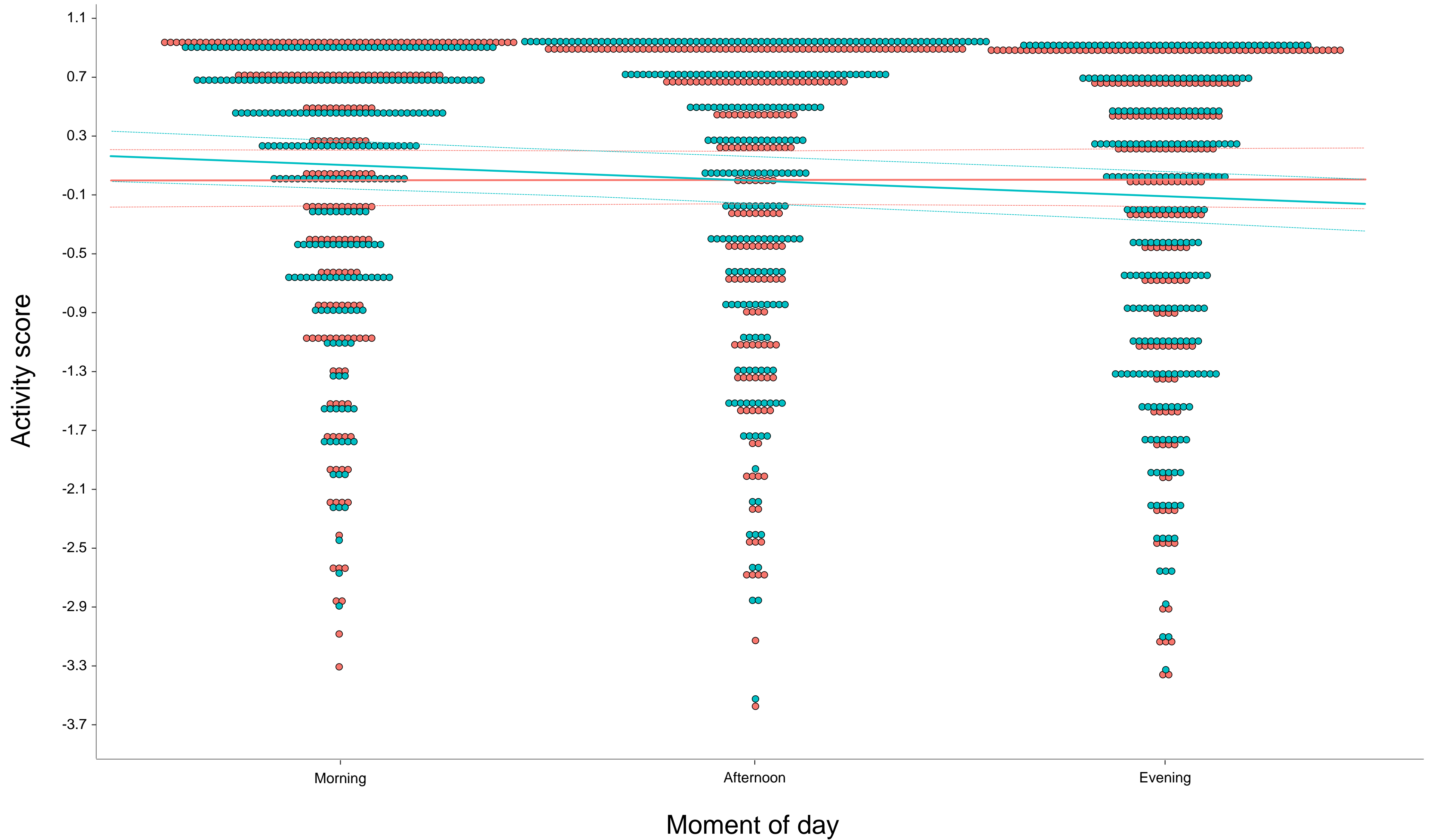

Supplement: Figure S2 — Average change in activity score over the course of day for males and females (including 95% confidence bands outlined with dashed lines), including raw data points. Males are indicated in blue, females are indicated in red. [file peerj-07-7177-s010.pdf]

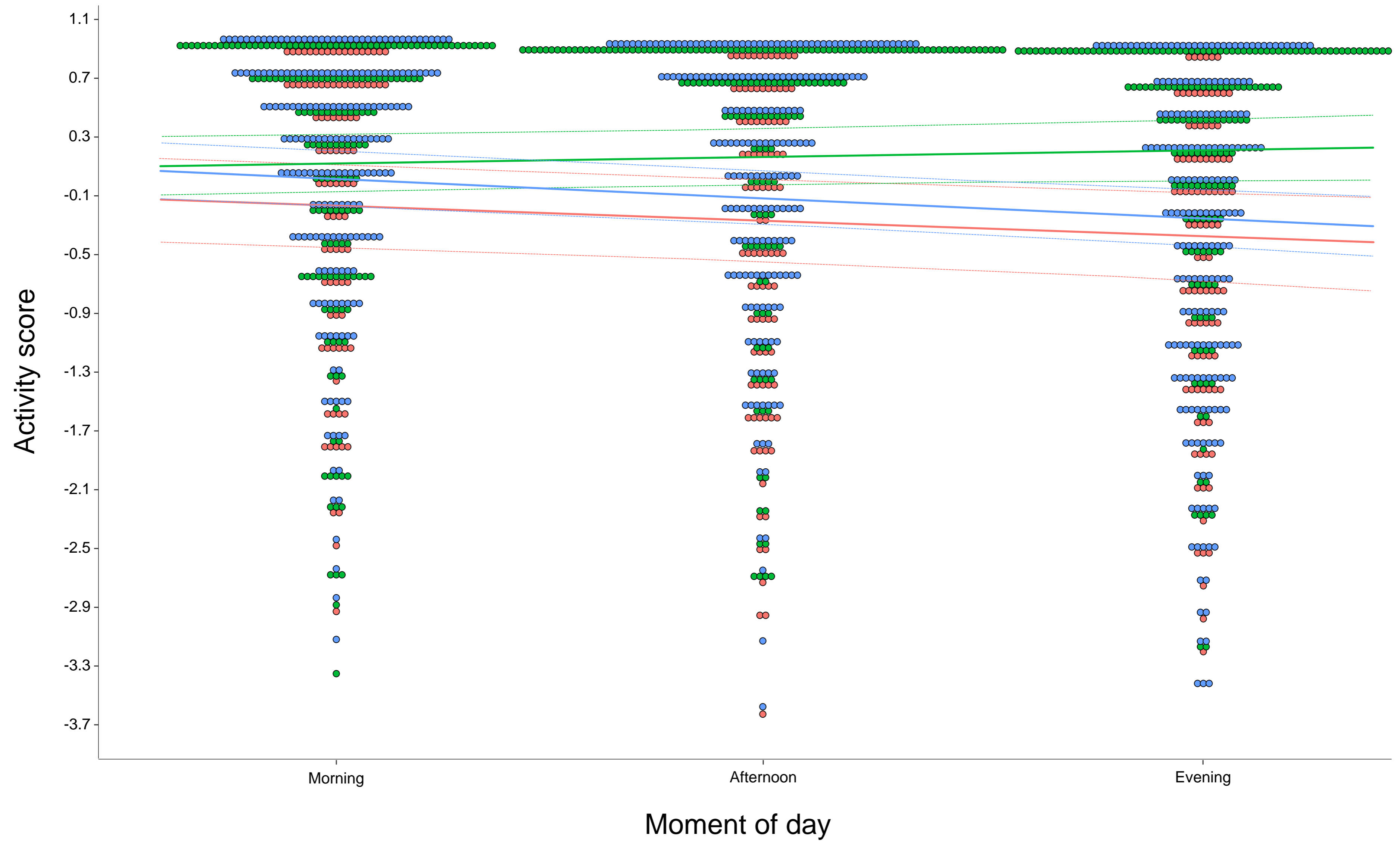

Supplement: Figure S3 — Average change in activity score over the course of day for each population type (including 95% confidence bands outlined with dashed lines), including raw data points. Long-lived population type is indicated in green, short-lived population type is indicated in blue and the short-lived GRZ population is indicated in red. [file peerj-07-7177-s011.pdf]

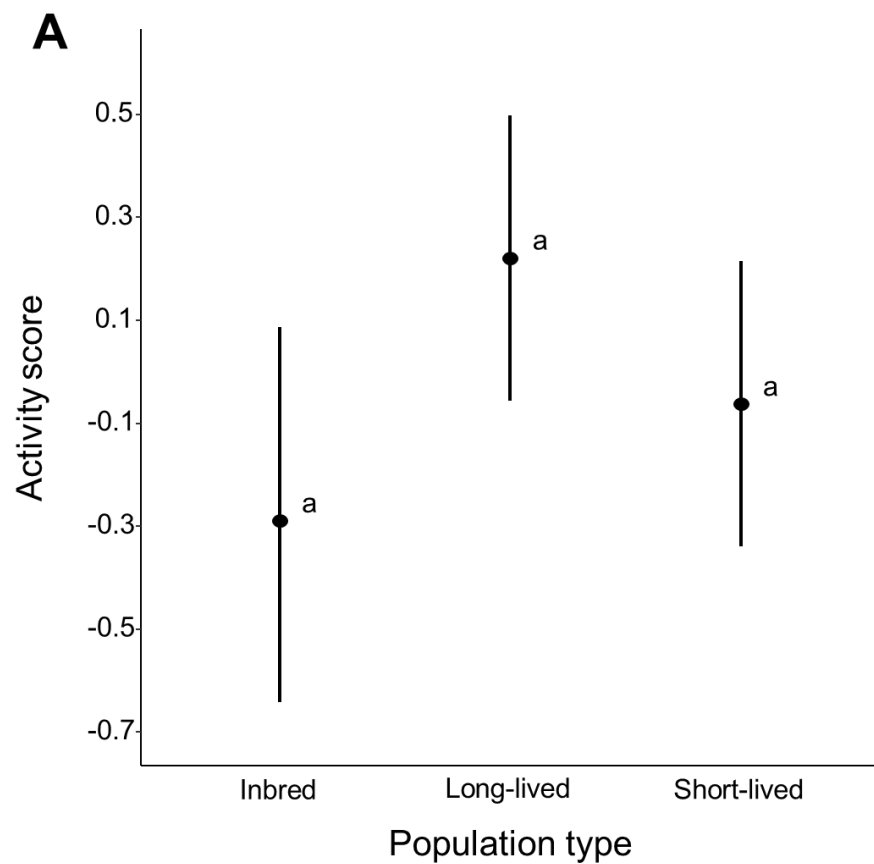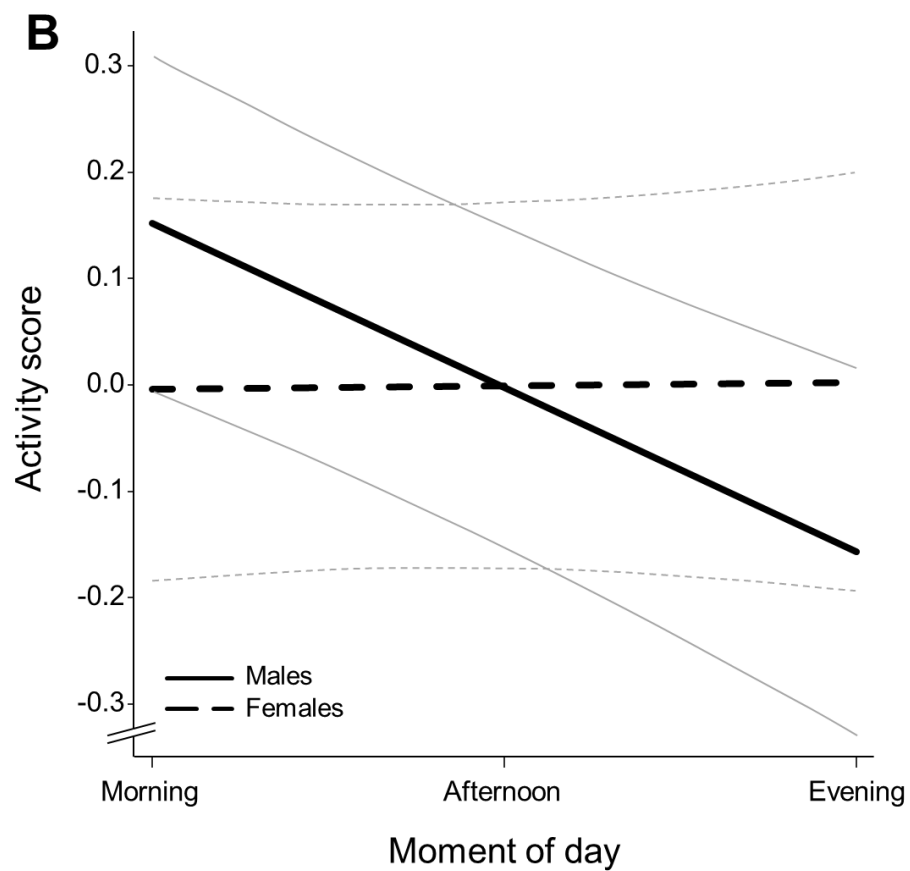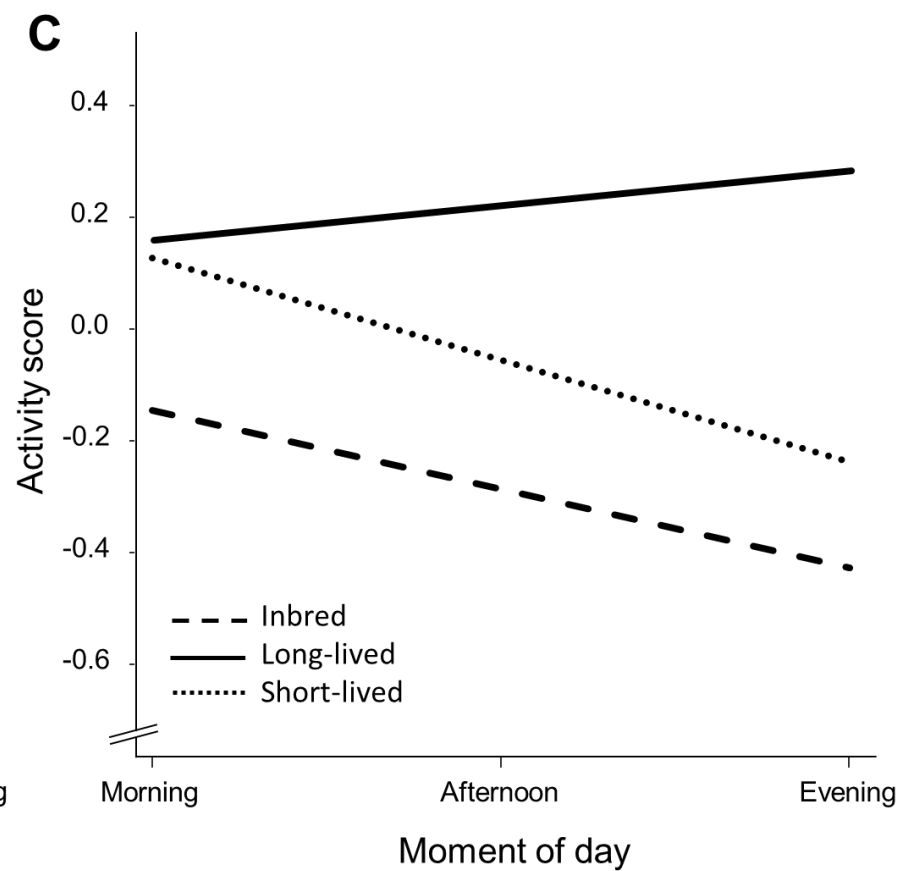

Supplement: Figure S4 — (A) Mean activity score for each population type (GRZ strain included). Whiskers delineate the upper and lower 95% confidence limit. Letters indicate significant differences based on Tukey-corrected post-hoc tests. (B) Average change in activity score over the course of day for males and females (including 95% confidence bands outlined in grey) and (C) for each population type. Slope coefficients and corresponding p-values are given in Table 2 for the long-lived and short-lived population types. Slope value of activity throughout the day for the GRZ strain is −0.131 (χ2 = 4.247, p = 0.039). [file peerj-07-7177-s012.pdf]

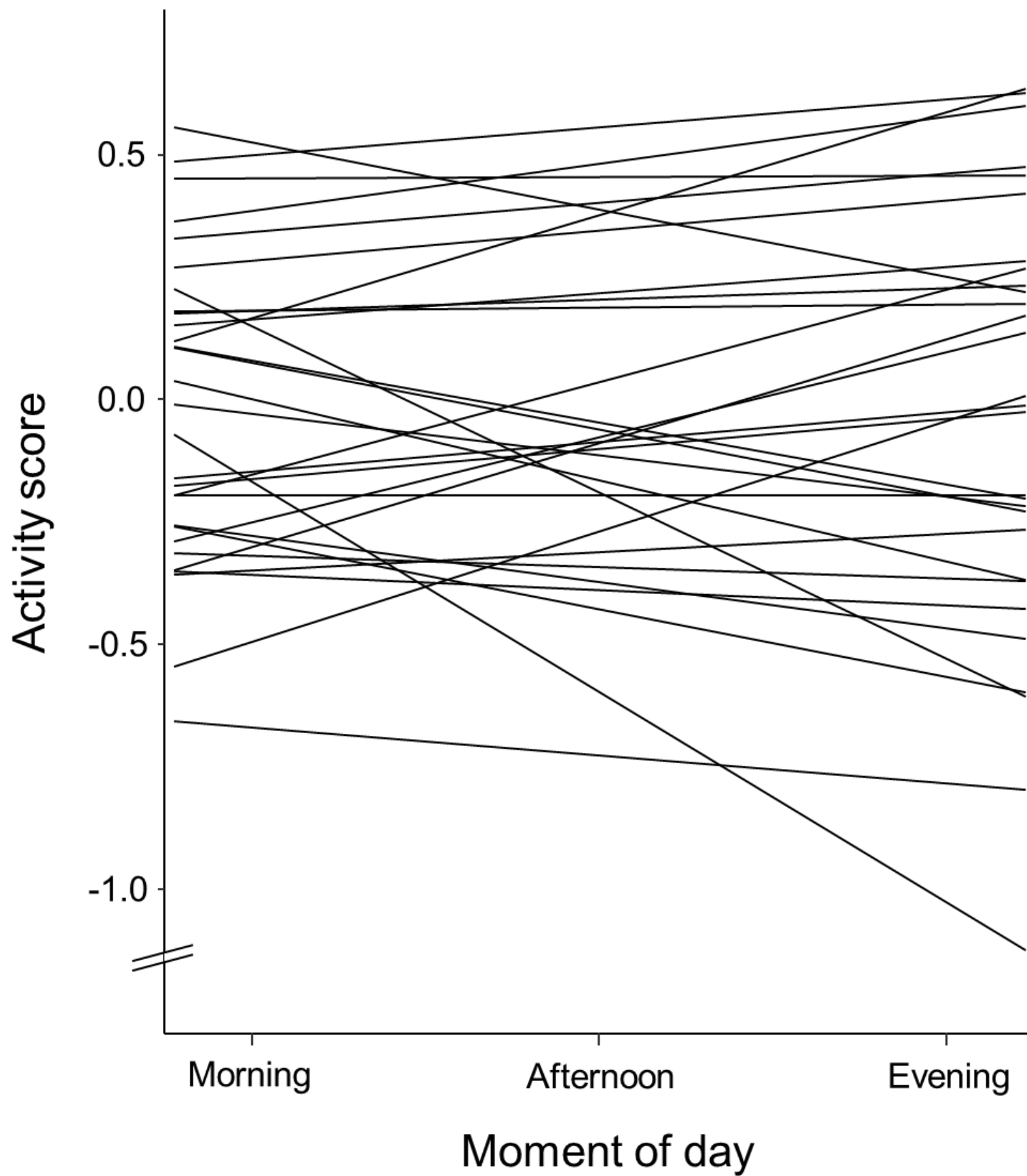

Supplement: Figure S5 — For clarity only 30 random individuals are shown. [file peerj-07-7177-s013.pdf]
